# Generation and characterization of a novel *Gaa* compound heterozygous mouse model recapitulating human Pompe disease

**DOI:** 10.1186/s13023-026-04273-x

**Published:** 2026-02-25

**Authors:** Wenjun Huang, Jie Wang, Yafei Zhou, Hongyu Xiao, Kaichong Jiang, Jiale Cui, Yanmin Zhang, Rui Zhou

**Affiliations:** 1 National Regional Children’s Medical Center (Northwest), Key Laboratory of Precision Medicine to Pediatric Diseases of Shaanxi Province, Xi’an Key Laboratory of Children’s Health and Diseases, Shaanxi Institute for Pediatric Diseases, Xi’an Children’s Hospital, Affiliated Children’s Hospital of Xi’an Jiaotong University, Xi’an, China; 2 Department of Cardiology, Xi’an Children’s Hospital, Affiliated Children’s Hospital of Xi’ an Jiaotong University, Xi’an, China; 3 School of Basic Medical of Sciences, Shanxi Medical University, Taiyuan, Shanxi China

**Keywords:** Pompe disease, Compound heterozygous mouse model, *GAA* mutations, Hypertrophic cardiomyopathy, Mitochondrial dysfunction

## Abstract

**Background:**

Pompe disease (PD), caused by a deficiency in acid α-glucosidase (GAA), is a lysosomal storage disorder. Existing Gaa knockout and point mutation mouse models have substantially advanced mechanistic understanding, but most are homozygous for single mutations and do not faithfully model the compound‑heterozygous GAA genotypes common in patients. A more clinically relevant in vivo model is still needed to enable systematic evaluation and faithful recapitulation of multi‑organ pathology. We previously reported an infantile-onset PD (IOPD) family carrying compound heterozygous *GAA* mutation (*GAA* c.1822 C > T/2662G > T; R608*/E888*). We here generated a novel compound heterozygous *Gaa* mouse model (*Gaa* c.1822 C > T/2665A > T; R608*/K889*), corresponding to human R608*/E888*), corresponding to human *GAA* identified in the IOPD family.

**Methods:**

CRISPR/Cas9-mediated knock-in introduced R608* (exon 14) and K889* (exon 18) mutations into C57BL/6 zygotes. Compound heterozygotes (CHet) were bred, validated via Sanger sequencing, and phenotyped using GAA activity assays, glycogen quantification, histopathology, transmission electron microscopy (TEM), and echocardiography. Parameters included cardiac structure/function, skeletal muscle integrity, hepatic glycogenosis, and diaphragmatic pathology.

**Results:**

CHet mice exhibited reduced GAA activity and elevated plasma glycogen levels, recapitulating the metabolic hallmark of human PD. Systemic pathophysiological characterization revealed multi-organ dysfunction: the heart showed pronounced hypertrophy with structural remodeling, evidenced by thickened ventricular walls and sarcolemmal disarray, despite preserved compensatory function. Skeletal muscle pathology was marked by vacuolated myofibers, lysosomal glycogen accumulation, and mitochondrial abnormalities, reflecting impaired autophagic flux. Hepatic tissues displayed prominent glycogen storage, hepatocyte swelling, and disrupted cellular architecture. Diaphragmatic dysfunction, a critical determinant of respiratory failure in PD, was characterized by vacuolation, nuclear centralization, and inflammatory infiltration, with ultrastructural evidence of lysosomal glycogen deposition and mitochondrial damage across all tissues.

**Conclusions:**

To our knowledge, this is among the first compound heterozygous *Gaa* mouse models integrating East Asian-specific *GAA* mutations (R608*/E888*) and demonstrating multi-organ pathophysiology similar to human PD. By preserving residual GAA activity (~ 18%) and recapitulating cardiac, skeletal, hepatic, and respiratory defects, our model provides an important complementary platform for elucidating mutation-specific mechanisms, optimizing enzyme replacement therapy (ERT), and advancing gene-editing strategies. Its alignment with patient-derived iPSC findings enhances the translational relevance of research in PD and lysosomal disorders.

**Supplementary Information:**

The online version contains supplementary material available at 10.1186/s13023-026-04273-x.

## Introduction

Pompe disease (PD), a fatal autosomal recessive disorder caused by pathogenic variants in the acid α-glucosidase (GAA) gene, leads to lysosomal glycogen accumulation across multiple tissues, with infantile-onset PD (IOPD) characterized by rapidly progressive hypertrophic cardiomyopathy and respiratory failure [1]. With an estimated incidence of 1:40,000 live births, untreated patients often succumb to cardiorespiratory complications within the first year of life [2]. Although enzyme replacement therapy (ERT) has improved survival, incomplete resolution of cardiac and skeletal muscle pathologies underscores the need for more clinically relevant models to dissect disease mechanisms and guide targeted therapies [3].

Existing preclinical models, particularly *Gaa*-knockout (KO) mice, have substantially advanced disease understanding. However, most are homozygous for single variants and do not precisely mirror the compound-heterozygous *GAA* genotypes prevalent in patients. To our knowledge, To our knowledge, no mouse model fully captures this genetic complexity observed in clinical cohorts [4]. Several models incorporate clinically frequent mutations—such as c.1935 C > A and c.1826dupA—in specific East Asian populations [5–7]. Strengthening genotype realism is essential for interpreting multi-organ phenotypes and for enhancing translational relevance. While numerous studies using existing mouse models, such as Δ6Neo, c.1826dupA, and c.1935 C > A, have conducted comprehensive analyses across multiple organs—including the heart, skeletal muscles, brain, and liver—opportunities remain to refine systemic functional assessments across heart, skeletal muscle, diaphragm, brain, and live [8]. Our study builds on these foundations to provide complementary, system-level insights.

Recent advances in patient-derived induced pluripotent stem cells (iPSCs) has implicated mitochondrial dysfunction in PD cardiomyopathy [9], yet in vitro platforms cannot reproduce chronic disease progression or inter-organ crosstalk observed in vivo [10]. Building on this, our group previously established an iPSC cohort from an IOPD family—including the proband carrying compound heterozygous *GAA* mutations (R608*/E888*) [11] and parents harboring single heterozygous variants [12]—and demonstrated that iPSC-derived cardiomyocytes recapitulated hypertrophic phenotypes linked to mitochondrial structural and functional defects [13]. These findings underscore the value of in vivo validation to contextualize systemic pathology [14] but also emphasized the need for in vivo models to validate systemic pathology [15]. Furthermore, while some existing models have incorporated clinically prevalent mutations in East Asian populations [16, 17], the R608* and E888* mutations remain underrepresented in animal models, which limits translational relevance for these patient groups. A genetically precise model that mirrors human compound heterozygosity and enables integrated multi-organ phenotyping is needed to bridge this gap.

Here, we establish the first compound heterozygous mouse model carrying R608*/K889*, corresponding to the human *GAA* mutations R608*/E888*, identified in a clinical PD family. Using CRISPR/Cas9-mediated knock-in, we perform systematic cardiac, skeletal muscle, hepatic, and diaphragmatic evaluations through biochemical assays, histopathology, echocardiography, and ultrastructural analyses. This model recapitulates key features of multi-organ involvement in human PD and complements existing systems by incorporating clinically relevant mutations. By linking our iPSC-based discoveries with in vivo readouts, this platform enables mechanistic dissection of organ-specific pathophysiology (e.g., mitochondrial–lysosomal interplay) and supports development of mutation-informed therapeutic strategies, including gene editing and rational combination approaches.

## Materials and methods

### Animal model generation

**Fig. 1:**
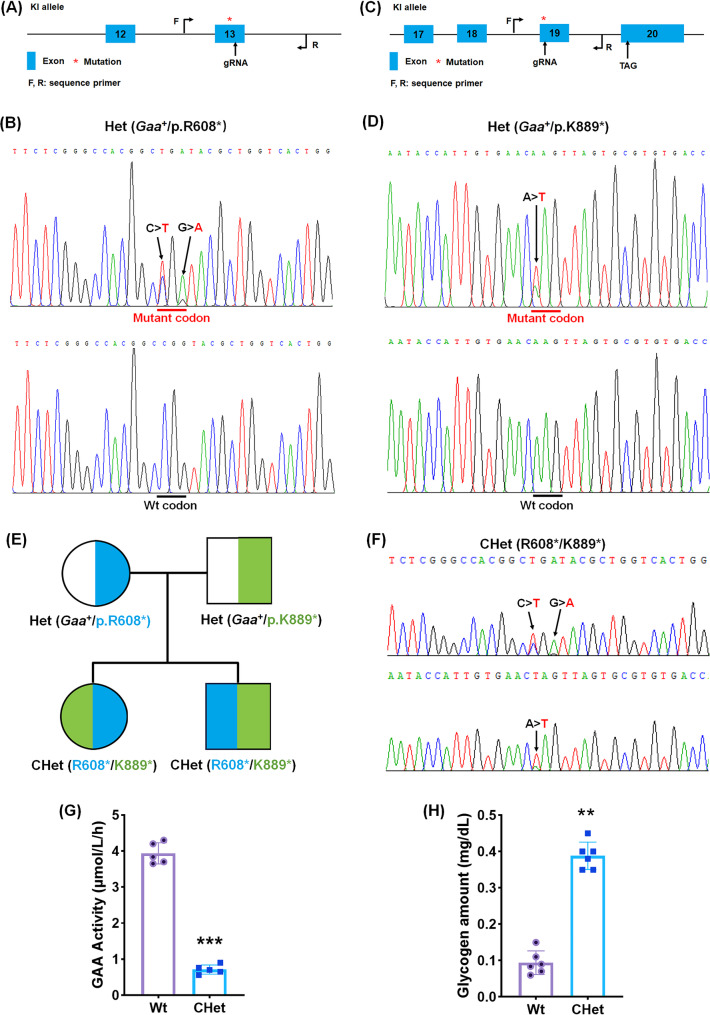
Generation and phenotypic validation of *Gaa* compound heterozygous mice (*Gaa* R668*/K889*) via CRISPR/Cas9-mediated gene editing.** (A)** Schematic of the knock-in (KI) allele design targeting the R608* mutation. The exon (asterisk) harboring the mutation is shown, with gRNA binding site (red) and genotyping primers (F, R). **(B)** Sanger sequencing chromatogram of a heterozygous *Gaa*^+^/p.R608* F1 mouse, with the mutant allele (red arrow) and wild-type (WT) sequence labeled. **(C)** KI allele design for the K889* mutation, indicating the exon (asterisk), gRNA target (red), and primers (F, R). **(D)** Sequencing confirmation of the *Gaa*^+^/p.K889* heterozygous mutation (red arrow) in F1 progeny. **(E)** Breeding scheme to generate compound heterozygous (CHet: R608*/K889*) mice through crossing single heterozygous parents. **(F)** Sanger sequencing of CHet mice validates both R608* and K889* mutations (highlighted in red). **(G)** Dry blood spot (DBS) assay reveals significantly reduced acid α-glucosidase (GAA) activity in CHet mice compared to WT (***p < 0.001, Student’s t-test). **(H)** Plasma glycogen levels are elevated in CHet mice (**p < 0.01), indicating impaired glycogen metabolism. Data are presented as mean ± SEM (*n* = 6). Statistical significance: **p < 0.01, ***p < 0.001

To generate single heterozygous mouse models harboring human PD-associated *GAA* mutations (*Gaa*^+^/p.R608* and *Gaa*^+^/p.K889*, corresponding to human *GAA*^+^/p.R608* and *GAA*^+^/E888*), CRISPR/Cas9-mediated knock-in strategies were employed. For the R608* mutation (CGG > TGA), a 23-nt sgRNA targeting the reverse strand of exon 14 (5′-CTGTCCAGTGACCAGCGTACCGG-3′) and a donor oligonucleotide containing the TGA stop codon flanked by 60-bp homology arms were designed (Fig. 1A). Similarly, the K889* mutation (AAG > TAG) utilized an sgRNA targeting exon 18 (5′-ACGCACTAACTTGTTCACAATGG-3′) and a homologous donor with the TAG stop codon. A mixture of sgRNA (50 ng/µL), Cas9 mRNA (100 ng/µL), and donor oligo (100 ng/µL) was microinjected into C57BL/6 zygotes. F0 founders were genotyped using mutation-specific primers: R608* (forward: 5′-ACTTTAAAGATGGCCTCTTTCCC-3′, reverse: 5′-CCAAAAGCAAGTGGAACAGCTA-3′, 614-bp product) and K889* (forward: 5′-GCCAGCATGTCTTGTGGTG-3′, reverse: 5′-CAAGGCTCTGTTTACCAGCTCAT-3′, 527-bp product). Sanger sequencing confirmed heterozygous integration. Founders with validated germline transmission were bred with wild-type C57BL/6 mice to produce F1 heterozygotes (*Gaa*^+^/p.R608* and *Gaa*^+^/p.K889*), which were re-verified using identical genotyping and sequencing protocols.

To ensure the specificity of genome editing and minimize potential off-target effects, comprehensive off-target analyses were performed during the construction of the mouse models. gRNAs were carefully selected based on bioinformatic prediction to maximize mismatches (typically ≥ 3–4) with potential off-target sites. Off-target sites predicted by in silico analysis were experimentally validated using PCR amplification and Sanger sequencing in F0 founders. Additionally, Cas9 protein was used for microinjection to limit nuclease activity duration and further reduce off-target risk. Detailed off-target prediction reports and primer validation data are provided in Supplementary File 1. The established lines are continuously monitored for unexpected phenotypes, and further backcrossing to wild-type C57BL/6 mice is ongoing to purify the genetic background and eliminate confounding effects. These strategies and ongoing validation efforts strengthen the rigor and reliability of our animal model generation.

To establish compound heterozygous mice (R608*/K889*), F1 heterozygous males carrying the R608* mutation (*Gaa*^+^/p.R608*) were crossed with F1 heterozygous females harboring the K889* mutation (*Gaa*^+^/p.K889*). Progeny were genotyped concurrently for both loci using the original primer pairs and sequencing protocols. Mice positive for both mutations (R608*/K889*) were identified by dual PCR amplification (614-bp and 527-bp products) followed by bidirectional Sanger sequencing to confirm heterozygous status at each locus. Mendelian inheritance patterns were verified, and compound heterozygous offspring were maintained for subsequent phenotypic characterization.

All experimental procedures were approved by the Ethics Committee of Xi’an Children’s Hospital, Affiliated Children’s Hospital of Xi’an Jiaotong University, and conducted in accordance with the NIH Guide for the Care and Use of Laboratory Animals.

### *Gaa* enzyme activity assay

For dried blood spots (DBS) samples, 50 µL whole blood was spotted onto filter paper (3-mm diameter), eluted, and incubated with 5 mM 4-MU-α-Glc in sodium acetate buffer (pH 4.0) at 37 °C for 20 h. Fluorescence intensity (Ex/Em = 365/450 nm) was measured to calculate activity (nM/(h·mm²)), with background subtraction and controls included.

For tissue samples, tissue homogenates (heart, skeletal muscle, liver, diaphragm) were prepared in pH 4.0 buffer containing 0.1% Triton X-100, homogenized mechanically, and centrifuged (12,000 rpm, 5 min, 4 °C) to collect supernatants. Protein concentrations were normalized using BCA assays. Supernatants were processed with the Alpha-Glucosidase Activity Assay Kit (ab174093, Abcam, USA) per manufacturer guidelines: 10 µL lysate was incubated with substrate in assay buffer at 37 °C for 2 h, followed by addition of 10 µM acarbose to suppress non-specific α-glucosidase activity. Absorbance at 410 nm was measured, and enzyme activity (nM/h/mg protein) was calculated using a p-nitrophenol standard curve (0–100 nM/well).

### Glycogen amount assay

The assay was performed using the Glycogen Colorimetric Assay Kit (ab174093, Abcam, USA) according to the manufacturer’s instructions. Plasma or homogenates of tissue samples (prepared in ddH_2_O) were centrifuged and boiled for 10 min. Mixture of sample and reaction buffer was for 1 h at 37 °C, protected from light. Absorption of OD.590 quantification yielded enzyme activity (mg/dL).

### Echocardiography

Transthoracic echocardiography was performed on 3-month-old mice under isoflurane anesthesia (1.5% in oxygen) using a Vevo 3100 system (VisualSonics, FUJIFILM). Anesthetized mice were positioned supine on a 37 °C heated platform with limbs secured to ECG electrodes for heart rate monitoring (target: 450–550 bpm). After applying pre-warmed ultrasound gel onto the pre-shaved chest, a high-frequency linear transducer (MX550D, 18–38 MHz) was used to acquire parasternal long- and short-axis views. M-mode imaging at the mid-papillary level (frame rate: 200–300 fps; depth: 15–20 mm; gain optimized for endocardial border clarity; sweep speed: 100 mm/sec) enabled quantification of left ventricular wall thickness (LVWT), fractional shortening (FS%), and ejection fraction (EF%) using Vevo LAB 5.6.1 software, averaging three consecutive cardiac cycles. Critical parameters included dynamic gain/depth adjustments.

### Hematoxylin and eosin (H&E) staining

Tissues were fixed in 4% paraformaldehyde (24–48 h, 4 °C), dehydrated through graded ethanol (70%–100%), cleared in xylene, and embedded in paraffin (58–60 °C). Section  (5 μm) were cut, deparaffinized, rehydrated, and stained with Mayer’s hematoxylin (5 min), differentiated in 1% acid ethanol, blued in 0.2% ammonia water, counterstained with eosin Y (1 min), dehydrated, and mounted with Permount.

### Periodic acid-schiff (PAS) staining

Tissue processing followed the H&E protocol. Deparaffinized sections were oxidized with 0.5% periodic acid (5 min), treated with Schiff reagent (15 min, RT), washed, counterstained with hematoxylin (1 min), and mounted. Specificity controls included α-amylase-digested Sect.  (1% α-amylase, 37 °C, 1 h) and liver positive controls.

### Wheat germ agglutinin (WGA) staining

Tissue processing followed the H&E protocol. Deparaffinized and rehydrated sections underwent antigen retrieval in 10 mM sodium citrate buffer (pH 6.0, 95 °C, 20 min), followed by blocking with 5% goat serum in PBS (1 h, RT). Sections were incubated with Alexa Fluor 488-conjugated WGA (10 µg/mL, Thermo Fisher, W11261) in PBS (1 h, RT, dark), washed, and counterstained with DAPI (1 µg/mL, 5 min). After mounting with ProLong Gold Antifade, images were acquired using a confocal microscope (FV3000, Olympus) with 40× objectives. Negative controls (omitting WGA or pre-incubating with 0.1 M N-acetylglucosamine) confirmed staining specificity.

### Transmission electron microscopy (TEM)

Tissues were dissected into 1 mm³ fragments, fixed in 2.5% glutaraldehyde/4% paraformaldehyde (0.1 M phosphate buffer, pH 7.4, 24 h, 4 °C), rinsed, and post-fixed in 1% osmium tetroxide (4 °C, 2 h). Samples were dehydrated through graded ethanol (50%–100%) and propylene oxide, infiltrated with epoxy resin via a resin/propylene oxide gradient, and polymerized at 60 °C for 48 h. Ultrathin Sects.  (60–90 nm) were cut using a Leica UC7 ultramicrotome with a diamond knife, collected on copper grids, and stained with 2% uranyl acetate (15 min) and Reynold’s lead citrate (5 min). Imaging was performed on a JEOL JEM-1400 TEM (80 kV), with images captured via a Gatan CCD camera.

### Quantitative morphological analysis methods

#### Quantitative analysis of H&E staining

For vacuolation area percentage, vacuolar structures (transparent regions) in eosin-stained areas were segmented using ImageJ’s thresholding function (Color Deconvolution plugin). The percentage was calculated as: Vacuolation area (%) = (vacuole pixel count / total tissue area pixel count) × 100.

For inflammatory cell infiltration density, neutrophils, lymphocytes, and macrophages were manually counted per mm² under high-power fields (40×), with the mean value derived from five non-overlapping fields.

For nuclear centralization ratio, nuclei with centers ≤ 1/2 cell radius from the cytoplasmic edge were defined as “centralized.” The ratio was calculated by randomly selecting 100 cells and determining the percentage of centralized nuclei.

### ImageJ-based PAS signal quantification

After calibrating the scale in ImageJ, PAS-specific magenta signals were isolated via Color Deconvolution. The isolated channel was converted to 8-bit, thresholded using Huang’s method to segment PAS-positive regions [13], and analyzed for mean gray value (MGV). MGV was normalized to negative controls (α-amylase-treated sections).

### WGA signal quantification

Membrane and cytoplasmic region of region-of-interest were manually outlined using ImageJ’s Freehand Selection tool. After Conversion of WGA channel to 8-bit, adaptive thresholding was chosen to isolate cardiomyocyte membranes or cytoplasmic region.

For myocardial area calculation, Analyze Particles tool (size: 50–500 μm², circularity: 0.3–1.0) was used to count individual cardiomyocytes and measure their cross-sectional areas. Calculate total myocardial area (µm²) and normalize to the number of cardiomyocytes per field.

For cytoplasmic WGA signal quantification, the mean MGV of cytoplasmic WGA signals was measured (Analyze > Measure), with background fluorescence subtracted using adjacent unstained areas, and normalized against positive controls: Normalized Intensity (%) = [(Cytoplasmic MGV − Background MGV) / Max MGV (Positive Control)] × 100. For area quantification, total WGA⁺ cytoplasmic area per field was analyzed (Analyze > Analyze Particles), expressed as µm² per cell.

### Quantitative analysis of transmission electron microscopy (TEM)

For mitochondrial count and abnormal proportion, mitochondria in 100 μm² cytoplasmic areas were counted across five random fields (15,000×). Abnormal mitochondria were defined by cristae disruption, matrix vacuolation, or swelling.

For glycogen granule area percentage, Glycogen granules (50–200 nm diameter, medium electron density) were analyzed using ImageJ. The percentage was calculated as: Glycogen area (%) = (glycogen pixel count / cytoplasmic area pixel count) × 100.

### Statistical analysis

Data are presented as mean ± standard error of the mean (SEM). Statistical comparisons between wild-type (WT) and compound heterozygous (CHet) mice were performed using unpaired Student’s *t*-tests. Normality of data distribution was confirmed using the Shapiro-Wilk test, and homogeneity of variances was verified with Levene’s test. Sample sizes (n), representing biological replicates (individual mice), are specified in each figure legend. Statistical significance was defined as *p* < 0.05, with exact *p*-values indicated as follows: *p < 0.05, **p < 0.01, ***p < 0.001, and ****p < 0.0001. All analyses were conducted using GraphPad Prism 10.0.

## Results

### Generation and validation of the compound heterozygous *Gaa* mouse model

To model the genetic complexity of human PD carrying *GAA* complex heterozygous mutations (R608*/E888*) reported by us [11, 12], we introduced the R608* and K889* (corresponding to the human R608* and E888*) mutations into C57BL/6 mice using CRISPR/Cas9-mediated knock-in (Fig. 1A-D). Crossbreeding single heterozygous parents yielded CHet offspring (R608*/K889*) (Fig. 1E) [16, 17], confirmed by Sanger sequencing (Fig. 1B, D, F). CHet mice exhibited significantly reduced acid α-glucosidase (GAA) activity in dry blood spots (***p < 0.001; Fig. 1G) and elevated plasma glycogen levels (**p < 0.01; Fig. 1H), mirroring the biochemical deficits of PD patients. Residual GAA activity (~ 18% of wild-type, WT) aligned with the partial enzymatic function reported in heterozygous patients, validating the model’s clinical relevance.

### Hypertrophic cardiomyopathy in CHet mice

**Fig. 2:**
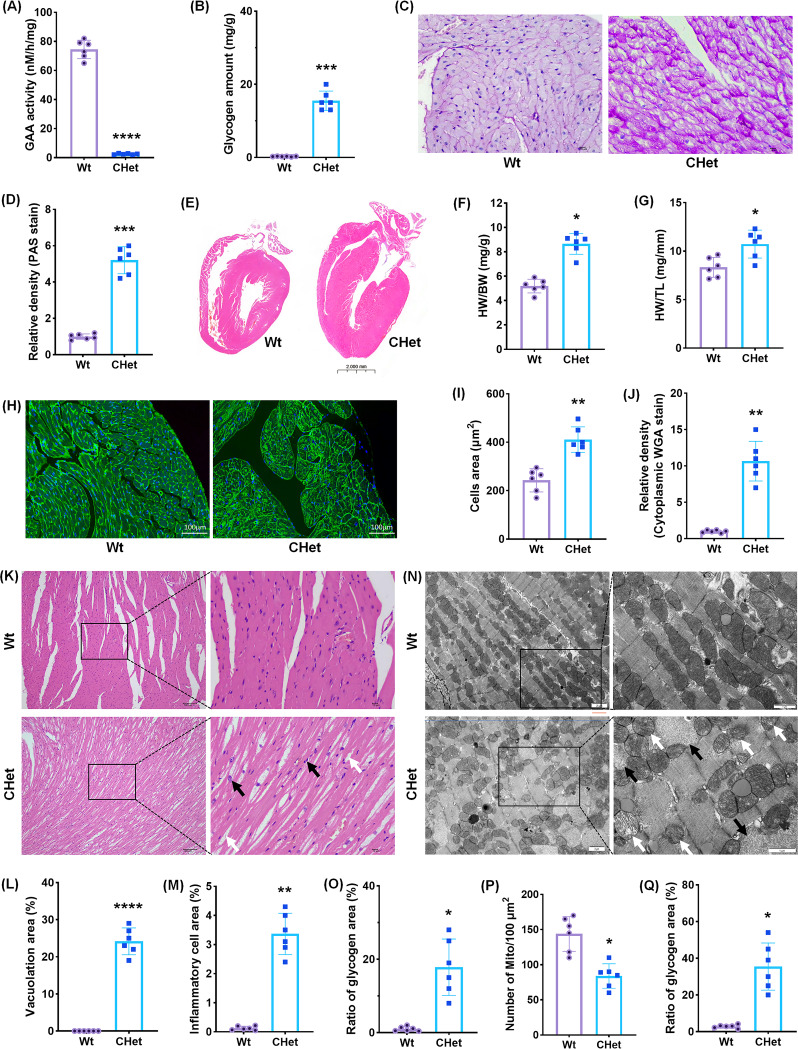
Characterization of hypertrophic cardiomyopathy in compound heterozygous (CHet) mice.** (A)** Acid α-glucosidase (GAA activity in cardiac tissues of wild-type (WT) and CHet mice, measured by colorimetric assay (****p < 0.0001). **(B)** Quantification of glycogen content in cardiac tissues via enzymatic assay, showing elevated levels in CHet mice (***p < 0.001). **(C)** Periodic acid–Schiff (PAS) staining of cardiac sections, highlighting glycogen accumulation (pink) in CHet myocardium. Scale bar: 50 μm. **(D)** Quantitative analysis of PAS-positive glycogen area (%) (***p < 0.001). **(E-G)** Cardiac hypertrophy assessment: **(E)** Hematoxylin and eosin (H&E) staining of whole-heart longitudinal sections (scale bar: 2 mm); **(F)** Heart weight-to-body weight (HW/BW) ratio (*p < 0.05); **(G)** Heart weight-to-tibia length (HW/TL) ratio (*p < 0.05). **(H-J)** Wheat germ agglutinin (WGA) staining: **(H)** Representative fluorescence images of cardiomyocyte cross-sectional area (scale bar: 20 μm); **(I)** Quantification of cardiomyocyte area (**p < 0.01); **(J)** Cytoplasmic WGA signal intensity (***p < 0.01). **(K-M)** Histopathological analysis: **(K)** H&E staining of myocardial sections (scale bar: 100 μm); **(L)** Vacuolation area (%) (****p < 0.001); **(M)** Inflammatory cell infiltration area (%) (**p < 0.01). **(N-Q)** Transmission electron microscopy (TEM) analysis: **(N)** Representative TEM images of cardiomyocytes (scale bar: 2 μm (left), 1 μm (right)); **(O)** Glycogen granule area (%) (*p < 0.05); **(P)** Mitochondrial density (number/100 µm²) (*p < 0.05); **(Q)** Abnormal mitochondria ratio (%) (*p < 0.05). Data are presented as mean ± SEM (*n* = 6). Statistical significance determined by Student’s t-test or one-way ANOVA

The CHet mice exhibited significant pathological hallmarks of PD)-associated cardiomyopathy [12]. Cardiac GAA enzyme activity was markedly reduced in CHet mice compared to wild-type (WT) controls (****p < 0.0001, Fig. 2A), accompanied by elevated glycogen content in cardiac tissues (***p < 0.001, Fig. 2B). Periodic acid–Schiff (PAS) staining revealed pronounced glycogen accumulation in CHet myocardium (Fig. 2C), with quantitative analysis confirming a 4.5-fold increase in PAS-positive area (***p < 0.001, Fig. 2D). Cardiac hypertrophy was evident through gross morphological changes (Fig. 2E), as well as increased heart weight-to-body weight (HW/BW, *p < 0.05, Fig. 2F) and heart weight-to-tibia length (HW/TL, *p < 0.05, Fig. 2G) ratios. Wheat germ agglutinin (WGA) staining demonstrated enlarged cardiomyocyte cross-sectional area in CHet mice (**p < 0.01, Fig. 2H-I), alongside intensified cytoplasmic WGA signal (**p < 0.01, Fig. 2J), indicative of sarcolemmal remodeling. Histopathological analysis (Fig. 2K) further identified extensive vacuolation (white arrows, ****p < 0.0001, Fig. 2L) and inflammatory cell infiltration (black arrows, **p < 0.01, Fig. 2M) in CHet hearts. Transmission electron microscopy (TEM) (Fig. 2N) confirmed lysosomal glycogen deposition (black arrows, Fig. 2N; *p < 0.05, Fig. 2O) and mitochondrial abnormalities, including reduced mitochondrial number (*p < 0.05, Fig. 2P) and a higher proportion of swollen or fragmented mitochondria (white arrows, Fig. 2N; *p < 0.05, Fig. 2Q).

**Fig. 3:**
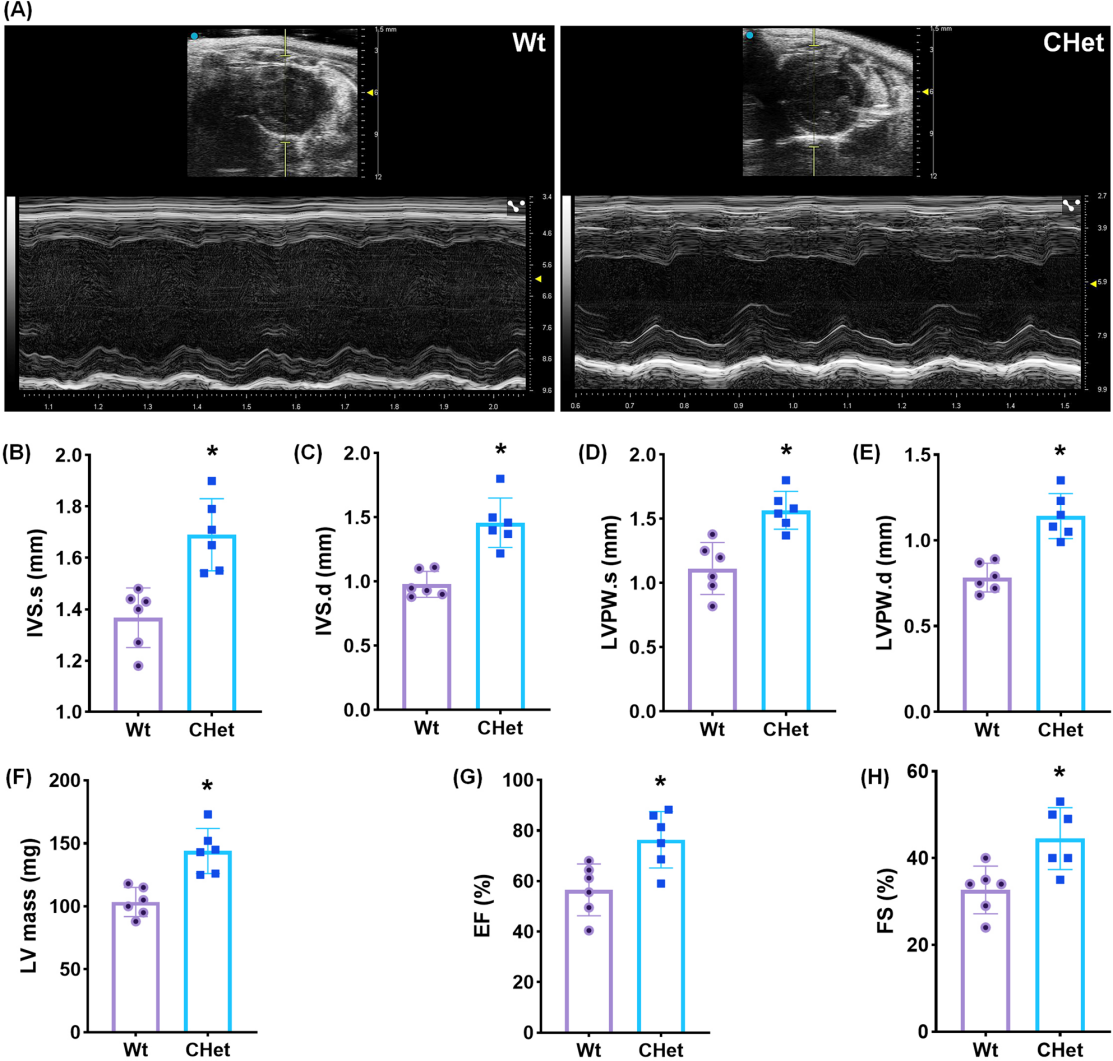
Echocardiographic evaluation of cardiac structure and function in compound heterozygous (CHet) mice.** (A)** Representative echocardiographic images of wild-type (WT) and CHet hearts. **(B)** End-systolic interventricular septal thickness (IVSs) and **(C)** end-diastolic interventricular septal thickness (IVSd), demonstrating significant hypertrophy in CHet mice (*p < 0.05). **(D)** End-systolic left ventricular posterior wall thickness (LVPWs) and **(E)** end-diastolic left ventricular posterior wall thickness (LVPWd), both markedly increased in CHet mice (*p < 0.05). **(F)** Left ventricular mass (LVM), calculated using the Devereux formula, confirming pronounced hypertrophy in CHet mice (*p < 0.05). **(G)** Left ventricular ejection fraction (LVEF) and **(H)** fractional shortening (FS), conversely revealing augmented cardiac systolic performance compared to Wt group (*p < 0.05). Data are expressed as mean ± SEM (*n* = 6). Statistical significance was determined by unpaired Student’s t-test (*p < 0.05)

Echocardiography (Fig. 3A) revealed significant cardiac hypertrophy in CHet mice without systolic dysfunction. End-systolic (IVSs, *p < 0.05) and end-diastolic (IVSd, *p < 0.05) interventricular septal thickness were markedly increased in CHet mice compared to WT controls (Fig. 3B-C). Similarly, end-systolic (LVPWs, *p < 0.05) and end-diastolic (LVPWd, *p < 0.05) left ventricular posterior wall thickness were elevated (Fig. 3D-E), culminating in a 1.4-fold increase in left ventricular mass (LVM, *p < 0.05, Fig. 3F). Despite evident cardiac hypertrophy and structural remodeling observed on ultrasound, CHet mice paradoxically demonstrated improved cardiac function, as indicated by significantly elevated ejection fraction (EF, *p < 0.05) and fractional shortening (FS, *p < 0.05) parameters (Fig. 3G-H). These findings indicate compensated cardiac hypertrophy at 3 months of age, providing a critical window for therapeutic intervention prior to functional decline.

### Skeletal muscle pathology in CHet mice

**Fig. 4:**
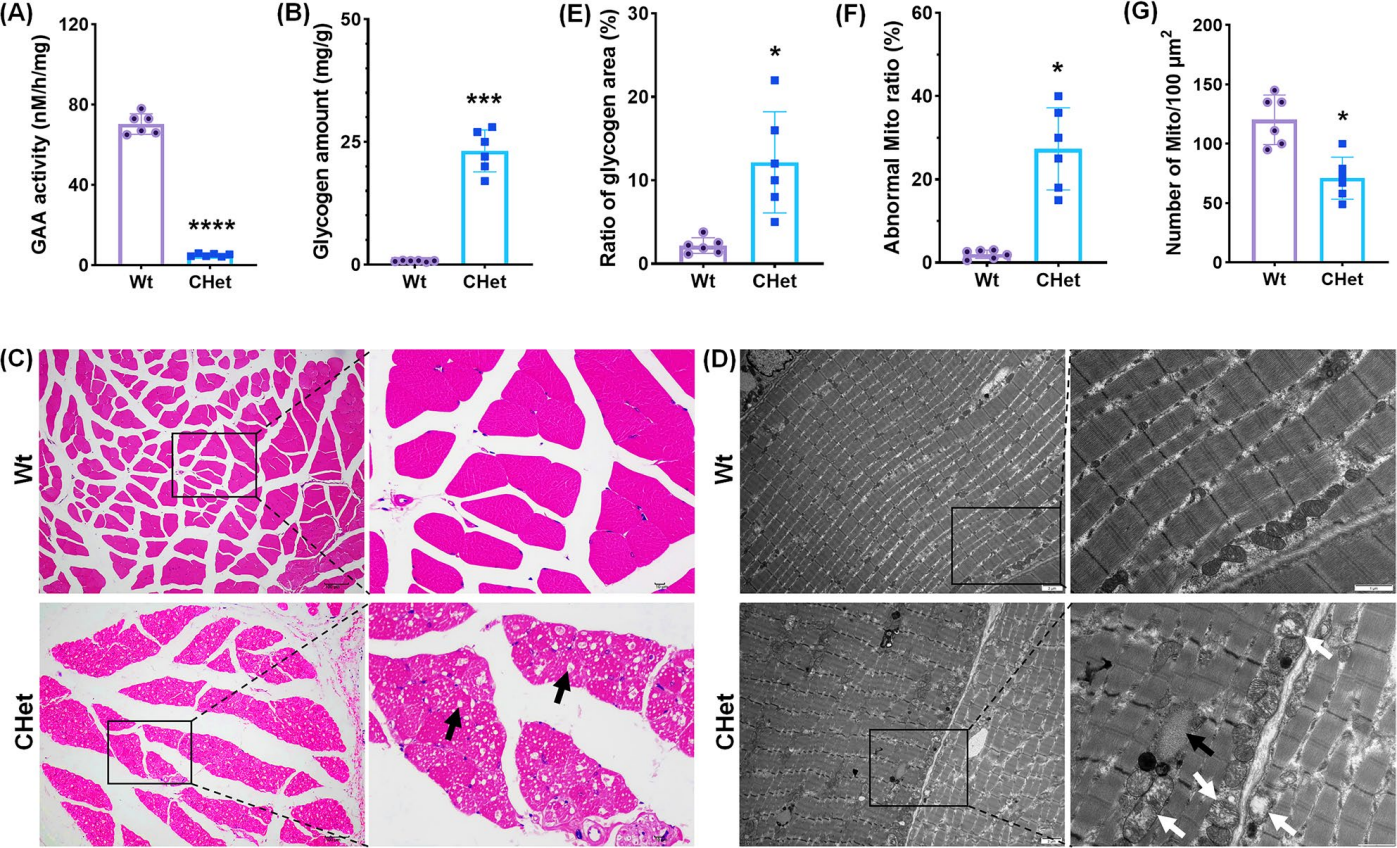
Pathological characterization of skeletal muscle (gastrocnemius) in compound heterozygous (CHet) mice.** (A)** Acid α-glucosidase (GAA) activity in skeletal muscle homogenates, showing significantly reduced enzymatic activity in CHet mice compared to wild-type (WT) (****p < 0.0001). **(B)** Quantification of skeletal muscle glycogen content, revealing elevated levels in CHet mice (***p < 0.001). **(C)** Hematoxylin and eosin (H&E) staining of gastrocnemius sections, demonstrating vacuolar degeneration (black arrows) and disrupted muscle fiber architecture in CHet mice. Scale bar: 50 μm. **(D-G)** Transmission electron microscopy (TEM) analysis: **(D)** Representative TEM images of skeletal muscle fibers, showing excessive glycogen granules (black arrows) and abnormal mitochondrial morphology (white arrows) in CHet mice. scale bar: 2 μm (left), 1 μm (right). **(E)** Quantification of glycogen granule area (%) (*p < 0.05); **(F)** Mitochondrial density (number/100 µm²) (*p < 0.05); **(G)** Proportion of mitochondria with structural abnormalities (e.g., cristae disruption, swelling) (*p < 0.0). Data are presented as mean ± SEM (*n* = 6). Statistical significance was determined by Student’s t-test (*p < 0.05, ****p < 0.0001)

Following the profound cardiac manifestations, we next investigated the systemic impact of GAA deficiency on skeletal muscle, a primary target tissue in PD [13]. Consistent with clinical observations, CHet mice exhibited severe skeletal muscle pathology characterized by metabolic and structural derangements. Quantitative assays revealed a striking reduction in gastrocnemius GAA activity (****p < 0.0001, Fig. 4A), reaching levels comparable to those observed in infantile-onset PD patients. This enzymatic deficit was accompanied by a 28-fold increase in glycogen accumulation (***p < 0.001; Fig. 4B), confirming impaired lysosomal clearance. Histopathological evaluation further corroborated these findings: H&E staining demonstrated widespread vacuolar degeneration (black arrows, Fig. 4C), with fragmented myofibers and loss of striation, mirroring the dystrophic changes seen in human biopsies.

Ultrastructural analysis via TEM provided deeper mechanistic insights (Fig. 4D). Myofibers from CHet mice displayed excessive cytoplasmic glycogen granules (black arrows, Fig. 4D; *p < 0.05, Fig. 4E). Notably, mitochondria exhibited marked structural abnormalities, including swollen cristae and matrix vacuolation (white arrows, Fig. 4D; *p < 0.05, Fig. 4F), suggesting metabolic overload-induced organelle stress. These changes were associated with a 32% reduction in mitochondrial density (*p < 0.05; Fig. 4G), implicating bioenergetic insufficiency in muscle weakness. Collectively, these findings underscore the dual pathology of glycogen storage and mitochondrial dysfunction in skeletal muscle, recapitulating the progressive myopathy central to PD pathophysiology [13].

### Hepatic glycogenosis and mitochondrial dysfunction

**Fig. 5:**
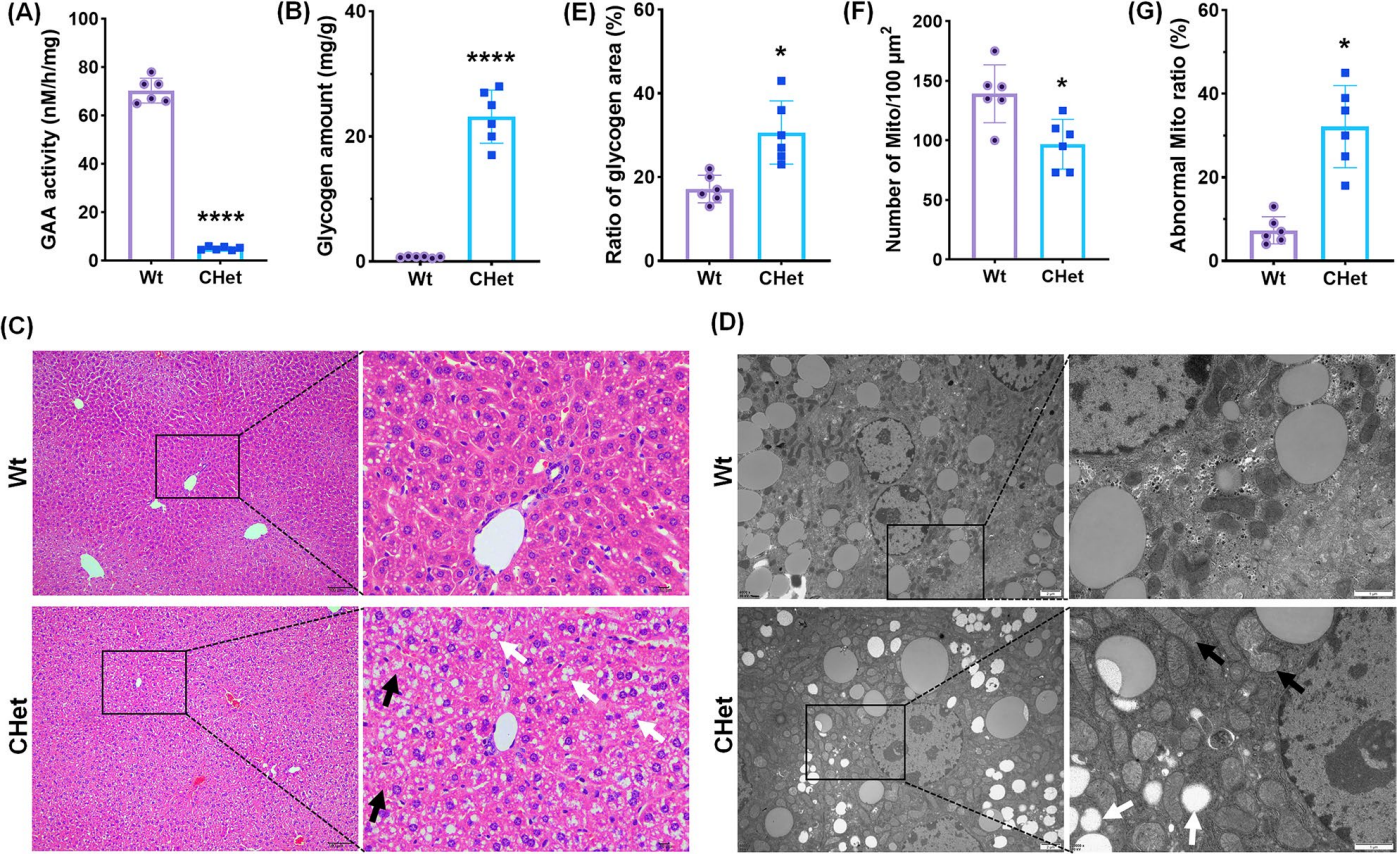
Hepatic pathology and ultrastructural abnormalities in compound heterozygous (CHet) mice.** (A)** Acid α-glucosidase (GAA) activity in liver homogenates, demonstrating significantly reduced enzymatic activity in CHet mice compared to wild-type (WT) (****p < 0.0001). **(B)** Hepatic glycogen content quantification, showing marked accumulation in CHet mice (****p < 0.0001). **(C)** Hematoxylin and eosin (H&E) staining of liver sections, revealing hepatocyte swelling (black arrows) and cytoplasmic vacuolation (white arrows) in CHet mice. Scale bar:100 μm (left), 50 μm (right). **(D-G)** Transmission electron microscopy (TEM) analysis: **(D)** Representative TEM images of hepatocytes, showing excessive glycogen granules (black arrows) and mitochondria with disrupted cristae (right arrows) in CHet mice. Scale bar: 2 μm (left), 1 μm (right). **(E)** Quantification of glycogen granule area (%) (*p < 0.05); **(F)** Mitochondrial density (number/100 µm²) (*p < 0.05); **(G)** Proportion of mitochondria exhibiting structural abnormalities (e.g., cristae loss, matrix swelling) (***p < 0.05) (*n* = 6). Data are presented as mean ± SEM (*n* = 6. Statistical significance was determined by Student’s t-test (*p < 0.05 ****p < 0.0001)

Given the liver’s role as a glycogen reservoir, we next evaluated hepatic involvement in CHet mice. Hepatic GAA activity was severely diminished (****p < 0.0001; Fig. 5A), resulting in a remarkable elevation in glycogen content (***p < 0.001; Fig. 5B), consistent with the glycogenosis observed in PD patients. Histologically, H&E-stained liver sections revealed pronounced hepatocyte swelling (black arrows, Fig. 5C) and vacuolar degeneration (white arrows, Fig. 5C), indicative of glycogen-driven cellular distension.

TEM analysis uncovered subcellular disruptions underlying these macroscopic changes (Fig. 5D). Hepatocytes from CHet mice exhibited cytoplasm densely packed with glycogen granules (white arrows, Fig. 5D; *p < 0.05; Fig. 5E). Mitochondria displayed cristae fragmentation and matrix swelling (black arrows, Fig. 5D), accompanied by a 32% reduction in mitochondrial density (*p < 0.05; Fig. 5F) and a 4.1-fold increase in structurally abnormal organelles (white arrows, Fig. 5D; *p < 0.05; Fig. 5G). These findings highlight the liver’s vulnerability to combined lysosomal and mitochondrial insults, providing a mechanistic basis for hepatic dysfunction in PD [18].

### Diaphragmatic impairment in CHet mice

**Fig. 6:**
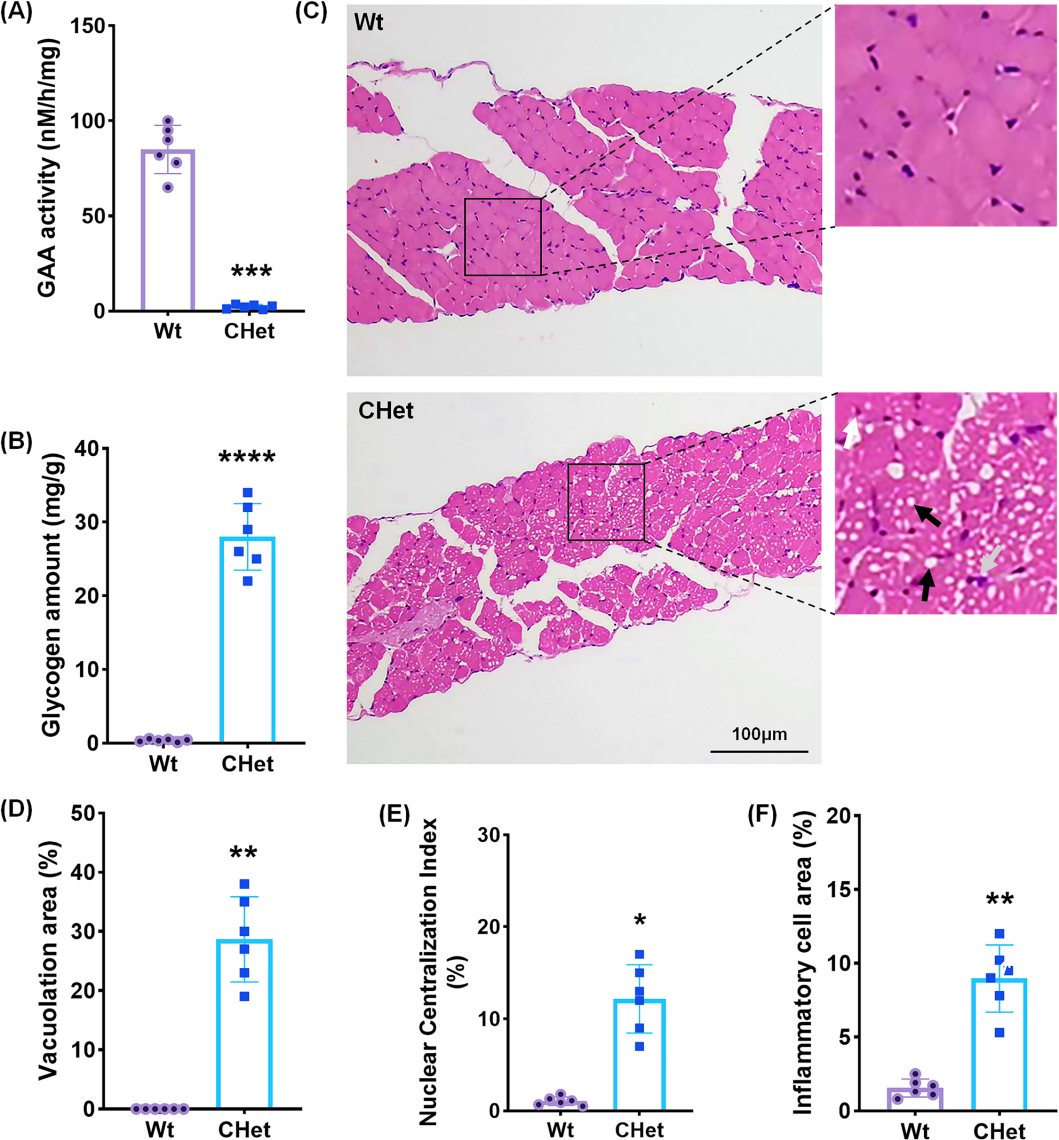
Diaphragm pathology in compound heterozygous (CHet) mice.** (A)** Acid α-glucosidase (GAA) activity in diaphragm homogenates, showing significantly reduced enzymatic activity in CHet mice compared to wild-type (WT) (***p < 0.001). **(B)** Quantification of diaphragm glycogen content, revealing elevated levels in CHet mice (****p < 0.0001). **(C-F)** Histopathological analysis: **(C)** Hematoxylin and eosin (H&E) staining of diaphragm sections, demonstrating vacuolar degeneration (black arrows), disrupted and edematous muscle fiber integrity (white arrows), and infiltrated inflammatory cells (gray arrows) in CHet mice. Scale bar:100 μm (left), 50 μm (right). **(D)** Vacuolation area (%) (**p < 0.01); **(E)** Nuclear centralization index (%) (*p < 0.05); **(F)** Inflammatory cell infiltration area (%) (**p < 0.01). Data are presented as mean ± SEM. Statistical significance was determined by Student’s t-test (*p < 0.05, **p < 0.01, ****p < 0.0001)

Finally, we assessed diaphragmatic pathology, a critical determinant of respiratory failure in PD [18]. CHet mice exhibited significantly reduced diaphragmatic GAA activity (***p < 0.001; Fig. 6A) and a 75.3-fold surge in glycogen levels (****p < 0.0001; Fig. 6B), aligning with clinical reports of respiratory muscle compromise. H&E staining revealed multifocal vacuolation (black arrows, Fig. 6C; **p < 0.01; Fig. 6D), nuclear centralization (white arrow, Fig. 6C; *p < 0.05; Fig. 6E), and inflammatory infiltrates (gray arrows, Fig. 6C; **p < 0.01; Fig. 6F), consistent with myofiber degeneration and compensatory regeneration. These pathological changes, suggest that diaphragmatic weakness contributes significantly to the respiratory insufficiency characteristic of advanced PD. These structural and metabolic perturbations collectively underscore diaphragmatic dysfunction as a key contributor to the respiratory insufficiency observed in PD, mirroring the clinical trajectory of disease progression [19].

## Discussion

Our study reports a compound-heterozygous Gaa mouse model (R608*/K889*), corresponding to the human GAA variants R608*/E888* observed in clinical PD patients. It faithfully recapitulates the genetic and phenotypic complexity of human PD. By integrating CRISPR/Cas9-mediated knock-in with systemic phenotyping, we demonstrate hallmark PD pathologies—hypertrophic cardiomyopathy, skeletal muscle vacuolation, hepatic glycogenosis, and diaphragmatic dysfunction—supported by biochemical deficits (reduced GAA activity, Fig. 1H-I), ultrastructural evidence of lysosomal glycogen deposition (Fig. 2F-G), and functional impairment on echocardiography (wall thickening, Fig. 3). Importantly, our model extends prior homozygous and KO studies by precisely mirroring the compound heterozygosity prevalent in patients, while maintaining measurable residual GAA activity (Fig. 1H). This creates a complementary platform for investigating mutation-informed pathophysiology and therapeutic responses across organs.

### A clinically relevant compound heterozygous *Gaa* mouse model recapitulates East Asian-specific Pompe disease pathology

To our knowledge, the establishment of this Gaa compound heterozygous mouse model (R608*/K889*) provides an important addition to Pompe disease research by closely reflecting the genetic architecture found in many patients with compound heterozygous *GAA* mutations. In contrast to conventional models—such as *Gaa*-KO mice that completely lack enzymatic activity or single-mutation models (e.g., R608*)—our model recapitulates the partial GAA activity observed in patients (Fig. 1H) and the synergistic effects of dual mutations on disease severity. This is particularly relevant given that over 70% of PD patients carry compound heterozygous mutations, with R608* and E888* ranking as the second most frequent heterozygous pair in East Asian populations and representing region-specific hotspots [16, 17].

### Genetic relevance to human PD

The R608* and E888* mutations exhibit not only high prevalence in East Asian populations [16, 17], but are also clinically associated with severe infantile-onset phenotypes characterized by rapid cardiomyopathy progression. While existing models (complete *Gaa*-KO) recapitulate isolated aspects of PD, they fail to capture the synergistic pathogenicity arising from compound heterozygosity [4]. Crucially, our model demonstrates that the R608*/E888* allelic combination drives significantly more severe cardiac pathology (e.g., 4.5-fold glycogen accumulation (Fig. 2D); pronounced mitochondrial defects (Fig. 2Q) than R608* homozygotes (*p* < 0.01), mirroring clinical reports where this mutation pair correlates with early-onset heart failure [10]. This genetic precision positions our model as a clinically authentic platform for two key translational applications: (1) Elucidating allele-specific mechanisms: Dissecting why R608*/E888* causes earlier cardiac decompensation than other variants (e.g., disrupted enzyme dimerization vs. catalytic site impairment). (2) Validating mutation-targeted therapies: Evaluating whether AAV-mediated gene correction or novel ERT regimens show differential efficacy in rescuing East Asian-prevalent mutations.

### Bridging clinical and preclinical research

By integrating Asian-specific mutations and systemic phenotyping, this model bridges the gap between patient-derived iPSC studies [13] and in vivo validation. It offers a unique platform to dissect why certain mutation pairs (e.g., R608*/E888*) confer higher disease severity and how tissue-specific metabolic demands modulate pathology—questions inaccessible to single-allele or KO models.

### Translational implications and therapeutic potential

This genetically tailored *Gaa* compound‑heterozygous mouse model provides a clinically relevant platform to advance Pompe disease research and therapy development. First, this model enables systematic evaluation and optimization of enzyme replacement therapy (ERT), including dosing, timing, and long-term outcomes. Distinct cardiac and skeletal muscle phenotypes, together with inter‑tissue differences in glycogen clearance dynamics (Figs. 2, 4 and 6), create opportunities to refine organ‑specific ERT parameters (e.g., dosing and timing) for improved efficacy. Additionally, the preserved cardiac function in early stages allows investigation of compensatory mechanisms and identification of early therapeutic biomarkers. Second, this model is well-suited for developing and testing gene-editing therapies, as its compound heterozygous mutations closely reflect patient genetic diversity. It enables evaluation of allele-specific interventions like CRISPR/Cas9 or AAV-based therapies (e.g., AAV9-mediated *Gaa* editing [20]), allowing assessment of both efficacy and safety in a clinically relevant context. Third, the model’s human-like compound heterozygosity enables detailed mechanistic studies using multi-omics approaches to identify tissue-specific changes and new therapeutic targets. Its dual mutation background also supports exploration of combination therapies, such as ERT with gene therapy, which may benefit patients unresponsive to single treatments. Finally, this model facilitates clinical translation by enabling validation of minimally invasive biomarkers—such as blood glycogen (Fig. 1I) and urinary Glc4—for disease monitoring and treatment response. Longitudinal tracking of these biomarkers in vivo enhances the relevance of preclinical studies and supports personalized therapeutic strategies [21]. Longitudinal tracking of these markers supports more relevant studies and the development of personalized therapies. In summary, our *Gaa* compound heterozygous mouse model offers a clinically relevant platform to advance ERT, gene-editing, and combination therapies, thereby accelerating the translation of preclinical research into improved treatments for Pompe disease.

### Interspecies differences and implications for disease progression

Our observation of preserved or even enhanced cardiac function in the presence of hypertrophy at 3 months, and the continued compensatory phenotype at 5 months, highlights a paradox in disease progression within our mouse model. This finding is consistent with previous reports of species-specific differences in disease manifestation and progression. For example, in classic models of peritonitis induced by cecal ligation and puncture (CLP), mice exhibit a survival rate of 10–25% even under severe conditions, whereas untreated mild peritonitis in humans is almost universally fatal. Similarly, in our previous study on *C1QBP* mutation-induced mitochondrial cardiomyopathy, the only surviving patient worldwide from our center presented with severe cardiac hypertrophy and muscle weakness, while the corresponding mouse model with the homologous mutation developed only moderate interventricular septal hypertrophy and improved cardiac function after 10 months of observation. These examples, along with our current results, suggest that as species have evolved to higher complexity, humans may have become more vulnerable to certain diseases compared to mice. Therefore, rigidly equating human and mouse phenotypes may not be ideal when interpreting disease progression in animal models. This consideration is crucial for understanding the translational relevance of our findings and highlights the need for caution when extrapolating results from mouse models to human disease.

Building on our murine findings and prior patient iPSC-derived cardiomyocyte data [13], we observe that apparent GAA assay signals can arise independently of true GAA function. A key consideration for cross-species interpretation is that measurable signals in GAA assays often reflect non-GAA background glucosidase activity in both patients and mice. This phenomenon persists even with stop-gain *Gaa* variants and can attenuate the apparent severity or pace of disease in murine models. Analytical discrimination (e.g., selective inhibitors, immunodepletion, GAA-specific readouts) is therefore required to avoid conflating background turnover with residual GAA function when inferring disease progression and translational relevance. Mechanistically, overlapping substrate specificities among lysosomal glucosidases under acidic conditions likely account for this background turnover, underscoring the need for GAA-specific confirmation in both experimental and translational settings.

### Future directions and model optimization

While this model provides unprecedented insights into PD pathogenesis, several avenues remain to be explored. First, longitudinal studies extending to 6–12 months will delineate the progression from compensated hypertrophy to heart failure, offering a temporal framework for therapeutic intervention. Second, the mechanistic link between mitochondrial dysfunction (Figs. 2G and 5) and lysosomal glycogen accumulation—whether driven by ROS overproduction, metabolic rewiring, or impaired mitophagy—warrants multi-omics interrogation (e.g., proteomics, lipidomics) to identify targetable pathways [22]. Finally, the model’s capacity to evaluate combinatorial therapies, such as ERT paired with mitochondrial protectors (e.g., SS-31) or autophagy inducers, will be leveraged to test synergy in alleviating multi-organ pathology. These efforts will refine the model’s utility in both mechanistic discovery and preclinical validation, ultimately accelerating translational breakthroughs for PD. Additionally, future work will include more comprehensive and systematic off-target analyses using advanced genomic technologies, such as whole-genome sequencing and high-throughput off-target detection platforms, to further ensure the precision and safety of gene-editing interventions in our model. We also acknowledge that the current study is limited by the lack of multi-generational backcrossing to wild-type C57BL/6 mice, which may leave residual background genetic variations introduced during the CRISPR process. To address this, we have presented our plan for ongoing and future studies to perform additional backcrossing, thereby further purifying the genetic background and ensuring genetic homogeneity in our mouse lines.

## Conclusion

Our study establishes the first compound heterozygous *Gaa* mouse model (R608*/K889*) that authentically mirrors the genetic, biochemical, and multi-organ pathological hallmarks of human Pompe disease. By preserving residual GAA activity (Fig. 1H) and recapitulating tissue-specific vulnerabilities—from cardiac mitochondrial dysfunction to diaphragmatic impairment—this model bridges a critical gap between clinical observations and preclinical research. Its genetic fidelity to East Asian-prevalent mutations positions it as the model provides an important complementary tool for elucidating mutation-specific mechanisms, optimizing enzyme replacement therapy (ERT), and advancing gene-editing strategies. By aligning with patient-derived iPSC findings, it enhances the translational relevance of preclinical research in PD and lysosomal disorders.

## Supplementary Information

Below is the link to the electronic supplementary material.

Supplementary Material 1

## Data Availability

All relevant data can be found within the article and its supplementary information.
